# Plant Natural Compounds with Antibacterial Activity towards Common Pathogens of Pond-Cultured Channel Catfish (*Ictalurus punctatus*)

**DOI:** 10.3390/toxins2071676

**Published:** 2010-06-28

**Authors:** Kevin K. Schrader

**Affiliations:** United States Department of Agriculture, Agricultural Research Service, Natural Products Utilization Research Unit, National Center for Natural Products Research, Post Office Box 8048, University of Mississippi, 38677, USA; Email: kevin.schrader@ars.usda.gov; Tel.: 662-915-1144; Fax: 662-915-1035

**Keywords:** antibacterial, channel catfish, chelerythrine, columnaris, ellagic acid, enteric septicemia of catfish, β-glycyrrhetinic acid, sorgoleone, therapeutant, wogonin

## Abstract

The bacteria *Edwardsiella ictaluri* and *Flavobacterium columnare* cause enteric septicemia and columnaris disease, respectively, in channel catfish (*Ictalurus punctatus*). Natural therapeutants may provide an alternative to current management approaches used by producers. In this study, a rapid bioassay identified plant compounds as potential therapeutants. Chelerythrine chloride and ellagic acid were the most toxic toward *E. ictaluri*, with 24-h IC50 of 7.3 mg/L and 15.1 mg/L, respectively, and MIC of 2.1 mg/L and 6.5 mg/L, respectively. Chelerythrine chloride, ellagic acid, β-glycyrrhetinic acid, sorgoleone, and wogonin were the most toxic towards two genomovars of *F. columnare*, and wogonin had the strongest antibacterial activity (MIC = 0.3 mg/L).

## 1. Introduction

Enteric septicemia of catfish (ESC) is the leading cause of mortality in pond-raised channel catfish in the United States and is caused by the Gram-negative bacterium *Edwardsiella ictaluri* [1]. The second leading cause of mortality in pond-raised catfish is columnaris disease which is caused by the Gram-negative bacterium *Flavobacterium columnare* [2]. The use of antibiotic-laden feed is one management approach that catfish producers use in the treatment of ESC and columnaris disease. However, concerns about the development of antibiotic resistant strains of *E. ictaluri* and *F. columnare* from the use of these antibiotics and public concerns about the environmental impact from the use of antibiotic-laden feeds in agriculture make the future use of medicated feed in catfish aquaculture uncertain.

Another management approach in dealing with ESC and columnaris infection in catfish aquaculture is the use of therapeutants. Therapeutic chemicals have been used less commonly to manage ESC than columnaris disease. Potassium permanganate (KMnO_4_) and copper sulfate have been used to treat columnaris [3]. Recent research demonstrated that KMnO_4_ has a prophylactic value, but only a marginal therapeutic value once an infection of columnaris is established in channel catfish [4]. However, these chemicals have several drawbacks including the following: (1) their toxicity can be affected by water chemistry which makes efficacious application rates more difficult; (2) they are highly phytotoxic and their use may result in poisoning of the non-target phytoplankton community, with subsequent water quality deterioration (e.g., low dissolved oxygen levels); and (3) their broad-spectrum toxicity requires careful application so that catfish are not killed [5]. Chloramine-T, diquat, and hydrogen peroxide have also been investigated as bath treatments for columnaris disease in channel catfish, and diquat was the most effective in reducing the mortality of acutely infected catfish [6]. A more recent efficacy study conducted in tanks also found that diquat was effective in reducing the mortalities of catfish from an acute columnaris infection [7]. However, diquat is currently labeled for aquatic use as an herbicide only and would require additional testing and evaluation prior to United States Food and Drug Administration (USFDA) approval for use against columnaris. In addition, diquat may be too expensive for use in large catfish production ponds, and a reduction of efficacy could occur due to the binding of diquat to organic matter in the ponds. Currently, the only bath treatment approved by the USFDA for the treatment of external columnaris infection in channel catfish is the commercial product 35% PEROX-AID® which has an application dose as H_2_O_2_ of 50–75 mg/L for 60 minutes for channel catfish fingerlings and adults (http://www.fda.gov/AnimalVeterinary/DevelopmentApprovalProcess/Aquaculture/ucm132954.htm). However, this commercial product is not recommended for use in earthen ponds due to the rapid degradation of hydrogen peroxide in the presence of organic matter. Columnaris disease can also occur during episodes of ESC in channel catfish [8]. The efficacy of 35% PEROX-AID® against ESC is still unproven, and the USFDA has not approved 35% PEROX-AID® for the treatment of ESC.

Plants offer a very large and relatively unexplored source of phytochemicals for evaluation as pesticides and antimicrobials. Previous research found that extracts from clove (*Caryophyllus aromaticum* (Myrtaceae)) and jambolan (*Syzygyum joabolanum* (Myrtaceae)) contain certain compounds with toxicity against human pathogenic bacteria resistant to antibiotics [9]. In addition, various plant extracts were identified to possess antibacterial activity towards the common human pathogen *Staphyl ococcus aureus* [10,11]. While these examples demonstrate the potential of plants to provide antibacterial compounds, there has so far been very limited research in the discovery of compounds from plants with antibacterial activity towards fish pathogenic bacteria.

Previously, a rapid 96-well microplate bioassay developed by Schrader and Harries [12] has been utilized to evaluate natural compounds and crude extracts from plants and other organisms for antibacterial activity towards *E. ictaluri* and *F. columnare*. Subsequently, promising compounds have been identified; for example, tannic acid which is highly toxic towards *E. ictaluri* and *F. columnare* [13]. Additional research is needed to discover and develop other natural compounds (not antibiotics) from plants and other organisms for use as therapeutants to manage ESC and columnaris disease in catfish aquaculture. In this study, a variety of plant compounds were evaluated to determine their potential for use as antibacterial compounds against *E. ictaluri* and *F. columnare*.

## 2. Results and Discussion

**Figure 1:**
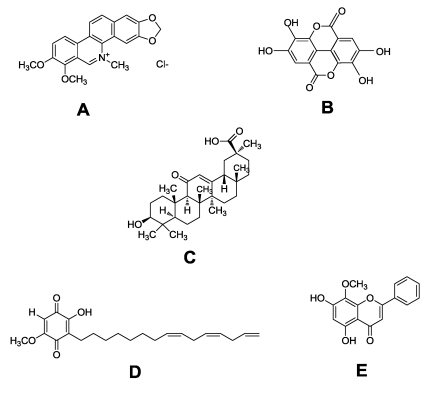
Chemical structures of: (a) Chelerythrine chloride; (b) Ellagic acid; (c) β-Glycyrrhetinic acid; (d) Sorgoleone; (e) Wogonin.

Chelerythrine chloride (Figure 1a) and ellagic acid (Figure 1b) had moderate to strong toxicity against *E. ictaluri* based upon 24-h IC50 and MIC results (Table 1). Chelerythrine chloride had significant toxicity, with a 24-h IC50 of 7.3 ± 0.8 mg/L and MIC of 2.1 ± 1.7 mg/L. Chelerythrine is a benzophenanthridine alkaloid that has been isolated from the extracts of several plants including *Chelidonium majus* (Papaveraceae) and *Zanthoxylum clava-herculis* (Rutaceae). Previous studies have cited the antibacterial activity of this alkaloid against methicillin-resistant *S. aureus* [14,15].

Ellagic acid also had significant toxicity toward *E. ictaluri*, with a 24-h IC50 of 15.1 ± 8.6 mg/L and MIC of 6.5 ± 5.0 mg/L. Ellagic acid is a phenolic compound found in many plants, and this phytochemical can be formed by the lactonization of hexahydroxydiphenic acid, a product of the hydrolysis of ellagitannin. Previous research by Chung *et al.* [16] found that ellagic acid was not strongly inhibitory towards the growth of 15 different bacterial species, though none of their test species were members of *Edwardsiella* or Flavobacteriaceae. Cai and Wu [17] also reported that ellagic acid did not contribute to the antimicrobial activity of the methanol crude extract of clove (*S. aromaticum*) towards the Gram-negative anaerobic bacteria *Porphyromonas gingivalis* and *Prevotella intermedia*. However, Thiem and Goślińska [18] attributed the significant antimicrobial activity of leaves of cloudberry (*Rubus chamaemorus* (Rosaceae)) towards several species of Gram-positive bacteria to the presence of ellagic acid in the leaves.

**Table 1:** Bioassay results of the toxicities of various plant compounds toward *Edwardsiella ictaluri*.

**Compound**	**24-h IC50^a^**	**MIC^b^**	**24-h IC50**		**MIC**	
	**(mg/L)**	**(mg/L)**	**RDCF^c^**	**RDCO^d^**	**RDCF^c^**	**RDCO^d^**
Acacetin	>284.3	>284.3	>2040.8	>3846.2	>1000.0	>1111.1
Allyl disulfide	>146.3	146.3	>2040.8	>3846.2	1000.0	1111.1
Apigenin	>270.2	>270.2	>2040.8	>3846.2	>1000.0	>1111.1
Apigenin 7-glucoside	>432.4	>432.4	>2040.8	>3846.2	>1000.0	>1111.1
Baicalein	243.2	27.0	1839.7	3461.5	100.0	111.1
Baicalin	>446.4	>446.4	>2040.8	>3846.2	>1000.0	>1111.1
Camphor	>152.2	>154.2	>2040.8	>3846.2	>1000.0	>1111.1
Chelerythrine chloride	7.3	2.1	38.8	73.1	10.0	11.1
Cichoriin	54.4	178.1	622.3	1173.1	1000.0	1111.1
*p*-Coumaric acid	>164.2	164.2	>2040.8	>3846.2	1000.0	1111.1
Cyanidin chloride	>287.2	>287.2	>2040.8	>3846.2	>1000.0	>1111.1
*p*-Cymene	>134.2	>134.2	>2040.8	>3846.2	>1000.0	>1111.1
Daidzein	>254.2	>254.2	>2040.8	>3846.2	>1000.0	>1111.1
Delphinidin chloride	101.6	>338.7	612.2	1153.9	>1000.0	>1111.1
Ellagic acid	15.1	6.5	83.3	143.6	10.0	10.6
Embelin	>294.4	>294.4	>2040.8	>3846.2	>1000.0	>1111.1
Emetine	>553.6	>553.6	>2040.8	>3846.2	>1000.0	>1111.1
Eucalyptol	>154.3	>154.3	>2040.8	>3846.2	>1000.0	>1111.1
Flavone	>222.2	>222.4	>2040.8	>3846.2	>1000.0	>1111.1
Gallocatechin	260.3	306.3	1734.7	3269.2	1000.0	1111.1
Genistein	>270.2	>270.2	>2040.8	>3846.2	>1000.0	>1111.1
Glycyrrhetinic acid (α)	>470.7	>470.7	>2040.8	>3846.2	>1000.0	>1111.1
Glycyrrhetinic acid (β)	>470.7	>470.7	>2040.8	>3846.2	>1000.0	>1111.1
Hyperforin	>536.8	>536.8	>2040.8	>3846.2	>1000.0	>1111.1
Inulin	>522.5	>522.5	>2040.8	>3846.2	>1000.0	>1111.1
Lavandulol	50.9	154.3	673.5	1269.2	1000.0	1111.1
(*S*)-Limonene	>136.2	>136.2	>2040.8	>3846.2	>1000.0	>1111.1
Myrcene	>136.2	>136.2	>2040.8	>3846.2	>1000.0	>1111.1
Myricetin	>318.2	318.2	>2040.8	>3846.2	1000.0	1111.1
Naringenin	>272.3	27.2	>2040.8	>3846.2	100.0	111.1
Pelargonidin chloride	85.9	>306.7	571.4	1076.9	>1000.0	>1111.1
Piceatannol	>244.3	>244.3	>2040.8	>3846.2	>1000.0	>1111.1
ß-Sitosterol	>414.7	>414.7	>2040.8	>3846.2	>1000.0	>1111.1
Sorgoleone	>358.0	>358.0	>2040.8	>3846.2	>1000.0	>1111.1
Stigmasterol	>412.7	>412.7	>2040.8	>3846.2	>1000.0	>1111.1
Vitexin	>432.4	>432.4	>2040.8	>3846.2	>1000.0	>1111.1
Wogonin	>284.2	>284.2	>2040.8	>3846.2	>1000.0	>1111.1
Zingiberone	>194.2	194.2	>2040.8	>3846.2	1000.0	1111.1

^a^ 24-h 50% inhibition concentration; ^b ^Minimum inhibitory concentration; ^c^ Relative to drug control florfenicol; values close to “1.0” indicate stronger antibacterial activity; ^d^ Relative to drug control oxytetracycline; values close to “1.0” indicate stronger antibacterial activity.

Based upon 24-h IC50 and MIC results, the following compounds possess moderate to strong activity against the two genomovars of *F. columnare* (ALM-00-173 and BioMed): baicalein, chelerythrine chloride, cichoriin, ellagic acid, flavone, genistein, β-glycyrrhetinic acid, piceatannol, sorgoleone, and wogonin (Table 2 and Table 3). Embelin, a benzoquinone-derivative isolated from *Embelia ribes* Myrsinaceae, was also moderately toxic toward *F. columnare* ALM-00-173 based upon MIC results (2.9 mg/L), but not as toxic toward *F. columnare* BioMed (MIC = 29.4 mg/L). Conversely, apigenin, a flavonoid found in many fruits and vegetables, was more toxic against *F. columnare* BioMed than *F. columnare* ALM-00-173 based upon MIC results of 2.7 mg/L and 27.0 mg/L, respectively. The genomovar II isolate *F. columnare* ALM-00-173 has been identified to be more pathogenic for channel catfish than genomovar I isolate *F. columnare* BioMed [19,20].

Baicalein (5,6,7-trihydroxyflavone) had similar toxicity against both genomovars of *F. columnare*. For *F. columnare* ALM-00-173 and *F. columnare* BioMed, the 24-h IC50 results were 8.2 ± 1.1 mg/L and 8.2 ± 0.1 mg/L, respectively, while MIC values were 14.9 ± 12.2 mg/L and 15.0 ± 12.3 mg/L, respectively. Baicalein is a flavonoid originally isolated from the roots of *Scutellaria baicalensis* (Lamiaceae), and it has been previously identified as possessing antibacterial activity [21]. In addition, baicalein will enhance the toxic activity of certain antibiotics, such as tetracycline, against bacteria (e.g., methicillin-resistant *Staphylococcus aureus*) [22].

Based upon 24-h IC50 results, cichoriin and flavone had moderate toxicity towards both isolates of *F. columnare*. Cichoriin has been isolated from the flowers of the plant *Cichorium intybus* (Asteraceae), commonly known as chicory [23]. Previous research fount that extracts of *C. intybus* had antibacterial activity [24]. Flavone (2-phenylchromone) is a pigment ubiquitous in plants and serves as a precursor molecule for other flavones such as apigenin.

Ellagic acid was similarly toxic towards *F. columnare* ALM-00-173 (24-h IC50 of 9.7 ± 3.4 mg/L and MIC of 3.0 ± 0 mg/L (Table 2)), and *F. columnare* BioMed (24-h IC50 10.3 ± 2.6 mg/L and MIC of 16.6 ± 19.2 mg/L (Table 3)). For the different test concentrations used in this study, ellagic acid was only bactericidal towards *F. columnare* BioMed and only at the highest concentration tested (MBC of 302.2 mg/L). None of the other compounds evaluated in this study were bactericidal at the concentrations tested. 

**Table 2:** Bioassay results of the toxicities of various plant compounds toward *Flavobacterium columnare* (ALM-00-173).

**Compound**	**24-h IC50^a^**	**MIC^b^**	**24-h IC50**		**MIC**	
	**(mg/L)**	**(mg/L)**	**RDCF^c^**	**RDCO^d^**	**RDCF^c^**	**RDCO^d^**
Acacetin	>284.3	>284.3	>518.1	>492.6	>1000.0	>1075.3
Allyl disulfide	>146.3	146.3	>518.1	>492.6	1000.0	1075.3
Apigenin	22.8	27.0	43.8	41.6	100.0	107.5
Apigenin 7-glucoside	216.2	>432.4	259.1	246.3	>1000.0	>1075.3
Baicalein	8.2	14.9	15.6	14.8	55.0	59.2
Baicalin	169.6	446.4	196.9	187.2	1000.0	1075.3
Camphor	>152.2	>154.2	>518.1	>492.6	1000.0	1075.3
Chelerythrine chloride	5.0	2.1	6.8	6.4	5.5	6.0
Cichoriin	12.4	17.8	36.0	34.3	100.0	107.5
*p*-Coumaric acid	>164.2	164.2	>518.1	>492.6	1000.0	1075.3
Cyanidin chloride	>322.7	>322.7	>518.1	>492.6	>1000.0	>1075.3
*p*-Cymene	>134.2	>134.2	>518.1	>492.6	>1000.0	>1075.3
Daidzein	>254.2	>254.2	>518.1	>492.6	>1000.0	>1075.3
Delphinidin chloride	54.2	>338.7	82.9	78.8	>1000.0	>1075.3
Ellagic acid	9.7	3.0	19.1	15.2	10.0	10.4
Embelin	13.0	2.9	22.8	21.7	100.0	10.8
Emetine	83.0	55.4	77.7	73.9	100.0	10.8
Eucalyptol	>154.3	>154.3	>518.1	>492.6	>1000.0	>1075.3
Flavone	6.7	22.2	15.5	14.8	100.0	107.5
Gallocatechin	55.1	30.6	93.3	88.7	100.0	10.8
Genistein	16.8	27.0	32.1	30.6	100.0	107.5
Glycyrrhetinic acid (α)	>470.7	>470.7	>518.1	>492.6	>1000.0	>1075.3
Glycyrrhetinic acid (β)	10.2	2.6	11.2	10.6	5.5	6.0
Hyperforin	>5.4	>5.4	>518.2	>4.9	>10.0	>10.8
Inulin	>522.5	522.5	>518.1	>492.6	1000.0	1075.3
Lavandulol	29.3	15.4	98.5	93.6	100.0	107.5
(*S*)-Limonene	>136.2	>136.2	>518.1	>492.6	>1000.0	>1075.3
Myrcene	>136.2	>136.2	>518.1	>492.6	>1000.0	>1075.3
Myricetin	28.6	31.8	46.6	44.3	100.0	107.5
Naringenin	46.3	27.2	88.1	83.7	100.0	107.5
Pelargonidin chloride	52.1	30.7	88.1	83.7	100.0	107.5
Piceatannol	9.7	24.4	20.5	19.5	100.0	107.5
ß-Sitosterol	>414.7	>414.7	>518.1	>492.6	>1000.0	>1075.3
Sorgoleone	9.0	3.6	13.0	12.3	10.0	10.8
Stigmasterol	>412.7	412.7	>518.1	>492.6	1000.0	1075.3
Vitexin	>432.4	>432.4	>518.1	>492.6	>1000.0	>1075.3
Wogonin	28.4	0.3	51.8	49.3	1.0	1.1
Zingiberone	>194.2	>194.2	>518.1	>492.6	>1000.0	>1075.3

^a^ 24-h 50% inhibition concentration; ^b^ Minimum inhibitory concentration; ^c^ Relative to drug control florfenicol; values close to “1.0” indicate stronger antibacterial activity; ^d^ Relative to drug control oxytetracycline; values close to “1.0” indicate stronger antibacterial activity.

**Table 3:** Bioassay results of the toxicities of various plant compounds toward *Flavobacterium columnare* (BioMed).

**Compound**	**24-h IC50^a^**	**MIC^b^**	**24-h IC50**		**MIC**	
	**(mg/L)**	**(mg/L)**	**RDCF^c^**	**RDCO^d^**	**RDCF^c^**	**RDCO^d^**
Acacetin	>284.3	>284.3	>423.7	>588.2	>1000.0	>1075.3
Allyl disulfide	>146.3	146.3	>423.7	>588.2	1000.0	1075.3
Apigenin	91.9	2.7	144.1	200.0	10.0	10.8
Apigenin 7-glucoside	255.1	>432.4	250.0	347.0	>1000.0	>1075.3
Baicalein	8.2	15.0	12.7	17.7	55.0	59.2
Baicalin	116.1	446.4	110.2	152.9	1000.0	107.5
Camphor	>152.2	>152.2	>423.7	>588.2	>1000.0	>1075.3
Chelerythrine chloride	7.9	2.1	8.7	12.1	5.5	6.0
Cichoriin	7.5	17.8	17.8	24.8	100.0	107.5
*p*-Coumaric acid	>164.2	164.2	>423.7	>588.2	1000.0	1075.3
Cyanidin chloride	242.0	>322.7	317.8	441.2	>1000.0	>1075.3
*p*-Cymene	>134.2	>134.2	>423.7	>588.2	>1000.0	>1075.3
Daidzein	>254.2	>254.2	>423.7	>588.2	>1000.0	>1075.3
Delphinidin chloride	135.5	338.7	169.5	235.3	1000.0	1075.3
Ellagic acid	10.3	16.6	14.1	17.8	55.0	58.8
Embelin	53.0	29.4	76.3	105.9	100.0	107.5
Emetine	121.8	55.4	93.2	129.4	100.0	107.5
Eucalyptol	>154.3	>154.3	>423.7	>588.2	>1000.0	>1075.3
Flavone	7.8	22.2	14.8	20.6	100.0	107.5
Gallocatechin	147.0	306.3	203.4	282.4	1000.0	1075.3
Genistein	16.2	1.5	25.5	35.3	5.5	6.0
Glycyrrhetinic acid (α)	>470.7	>470.7	>423.7	>588.2	>1000.0	>1075.3
Glycyrrhetinic acid (β)	13.7	47.1	12.2	17.1	100.0	107.5
Hyperforin	>5.4	>5.4	>4.2	>5.9	>10.0	>10.8
Inulin	>522.5	>522.5	>423.7	>588.2	>1000.0	>1075.3
Lavandulol	29.3	15.4	80.5	111.8	100.0	107.5
(*S*)-Limonene	>136.2	>136.2	>423.7	>588.2	>1000.0	>1075.3
Myrcene	>136.2	>136.2	>423.7	>588.2	>1000.0	>1075.3
Myricetin	>318.2	31.8	>423.7	>588.2	100.0	107.5
Naringenin	38.1	27.2	59.3	82.4	100.0	107.5
Pelargonidin chloride	116.5	>306.7	161.0	223.5	>1000.0	>1075.3
Piceatannol	8.7	24.4	15.1	20.9	100.0	107.5
ß-Sitosterol	>414.7	>414.7	>423.7	>588.2	>1000.0	>1075.3
Sorgoleone	9.3	3.6	11.1	15.3	10.0	10.8
Stigmasterol	>412.7	412.7	>423.7	>588.2	1000.0	1075.3
Vitexin	>432.4	>432.4	>423.7	>588.2	>1000.0	>1075.3
Wogonin	19.4	0.3	28.8	40.0	1.0	1.1
Zingiberone	>194.2	>194.2	>423.7	>588.2	>1000.0	>1075.3

^a^ 24-h 50% inhibition concentration; ^b ^Minimum inhibitory concentration; ^c ^Relative to drug control florfenicol; values close to “1.0” indicate stronger antibacterial activity; ^d^ Relative to drug control oxytetracycline; values close to “1.0” indicate stronger antibacterial activity.

Genistein was more toxic against *F. columnare* BioMed than *F. columnare* ALM-00-173 based upon MIC-RDC values of RDCF = 5.5 ± 4.5 and RDCO = 6.0 ± 4.9 for *F. columnare* BioMed while RDCF = 100.0 ± 0 and RDCO = 107.5 ± 0 for *F. columnare* ALM-00-173. Genistein is an isoflavone that has been identified in various plants including the roots of kudzu vine (*Pueraria lobata* (Fabaceae)) and soybean (*Glycine max* (Fabaceae)) [25]. Previous research identified the differential antibacterial activity of genistein towards various test bacterial species [26]. Another study suggested genistein to possess bacteriostatic activity rather than bactericidal activity based upon cell survival [27].

Interestingly, β-glycyrrhetinic acid (Figure 1c) was toxic towards both *F. columnare* isolates, though slightly more toxic against *F. columnare* ALM-00-173, while α-glycyrrhetinic acid showed no toxicity towards both isolates at the highest test concentrations used in this study. β-Glycyrrhetinic acid yielded 24-h IC50 results of 10.2 ± 1.7 mg/L and 13.7 ± 0 mg/L for *F. columnare* ALM-00-173 and *F. columnare* BioMed, respectively. The MIC results also indicated greater toxicity of β-glycyrrhetinic acid toward *F. columnare* ALM-00-173, with a MIC of 2.6 ± 2.1 mg/L compared to a MIC of 47.1 ± 0 mg/L for *F. columnare* BioMed. The MIC-RDC values also indicated strong toxicity of β-glycyrrhetinic acid toward *F. columnare* ALM-00-173 (MIC-RDCF = 5.5 ± 4.5 and MIC-RDCO = 6.0 ± 4.9). Glycyrrhetinic acid can be obtained from licorice, a root extract from *Glycyrrhiza glabra* Fabaceae. Previous research found β-glycyrrhetinic acid to inhibit the growth of the Gram-positive bacteria *Bacillus subtilis* and *Staphylococcus epidermidis*, but not the Gram-negative bacteria *Escherichia coli* and *Proteus vulgaris* [28]. In the same study, β-glycyrrhetinic acid also inhibited DNA, RNA, and protein synthesis in the Gram-positive bacteria.

The hydroxystilbene piceatannol was moderately toxic towards both isolates of *F. columnare* based upon MIC results of 9.7 ± 0.9 mg/L and 8.7 ± 2.3 mg/L for *F. columnare* ALM-00-173 and *F. columnare* BioMed, respectively. Piceatannol has been isolated from the plant *Mezoneuron cucullatum* (Leguminosae) and exhibited anti-carcinogenic properties [29]. 

Sorgoleone (2-hydroxy-5-methoxy-3-((8’*Z*,11’*Z*)-8’,11’,14’-pentadecatriene)-*p*-benzoquinone) (Figure 1d), an allelochemical produced by sorghum (*Sorghum bicolor* (Poaceae)), was similar in toxicity to both genomovars of *F. columnare* based upon 24-h IC50 and MIC results. The 24-h IC50 of sorgoleone for *F. columnare* ALM-00-173 and *F. columnare* BioMed were 9.0 ± 0.4 mg/L and 9.3 ± 0.7 mg/L, respectively, while the MIC was 3.6 ± 0 mg/L for both isolates. Preliminary research has indicated that sorgoleone can inhibit the growth of certain microorganisms found in the soil while also being used as a carbon source by other soil microflora [30,31]. 

Wogonin (5,7-dihydroxy-8-methoxyflavone) (Figure 1e) has also been isolated from the roots of *S. baicalensis* [32]. However, wogonin is recognized more for its therapeutic and protective effects against certain toxins than antibacterial properties [33,34]. In the current study, wogonin possessed the strongest antibacterial activity against both genomovars of *F. columnare* based upon a MIC of 0.3 ± 0 mg/L. The MIC-RDC results for wogonin of 1.0 ± 0 and 1.1 ± 0 for RDCF and RDCO, respectively, indicate essentially the same degree of antibacterial activity as the antibiotics florfenicol and oxytetracycline.

Overall, chelerythrine chloride, ellagic acid, β-glycyrrhetinic acid, sorgoleone, and wogonin appear to be the most promising of the active compounds evaluated against the genomovars *F. columnare* ALM-00-173 and *F. columnare* BioMed when collectively considering 24-h IC50 and MIC results. Although the objective of this study was to identify plant compounds that might be useful as therapeutants, some of the active compounds have little or no solubility in water, and, therefore, it would be difficult to efficacy test such a compound as a therapeutant in an aquatic environment. Chemical modification of the structure might be one method to impart water solubility, though toxic activity could be compromised. A potential alternative might be incorporation of the compound into the fish feed in order to determine efficacy. For example, β-glycyrrhetinic acid is insoluble in water and could possibly be added to the feed to determine any benefits in reducing columnaris infection in channel catfish. However, palatability of the feed after incorporation of the test compound is critical under such circumstances.

## 3. Materials and Methods

A culture of *E. ictaluri* (isolate S02-1039) was obtained from Mr. Tim Santucci (College of Veterinary Medicine, Mississippi State University, Stoneville, Mississippi), and cultures of two genomovars of *F. columnare* (BioMed (genomovar I) and ALM-00-173 (genomovar II)) were obtained from Dr. Covadonga Arias (Department of Fisheries and Allied Aquacultures, Auburn University, Alabama). In order to assure purity, *E. ictaluri* was maintained on 3.8% Mueller-Hinton (MH) agar plates (pH 7.3) (Becton, Dickinson and Company, Sparks, Maryland) while cultures of *F. columnare* isolates were maintained on modified Shieh agar plates (pH 7.2–7.4) [35]. Prior to conducting the bioassay, single colonies of the test cultures were used to prepare the assay culture materials as follows: (1) for *E. ictaluri*, 45 mL of 3.8% MH at 0.5 McFarland standard [12]; and (2) for *F. columnare*, each genomovar isolate was cultured separately in 75 mL of modified Shieh broth (18 h for BioMed and 24 h for ALM-00-173) at 29 ± 1 at 150 rpm on a rotary shaker (model C24KC; New Brunswick Scientific, Edison, New Jersey).

Compounds were evaluated for antibacterial activity using a rapid 96-well microplate bioassay and following the procedures for the growth assay portion of the rapid bioassay developed by Schrader and Harries [12]. Florfenicol and oxytetracycline HCl, antibiotics that can be used in medicated feed for catfish, were included as positive drug controls for each assay. Also, control wells (no test compound added) were included in each assay. Technical grade solvents were used to dissolve the test compounds (see Table 4). Final concentrations of test compounds in the microplate wells were 1.0, 10.0, 100.0, and 1,000.0 µM. Three replications were used for each dilution of each test compound and controls. Final results were converted to units of mg/L to allow comparison with previous studies.

**Table 4:** Compounds evaluated for toxicity towards *Edwardsiella ictaluri* and *Flavobacterium columnare*.

**Compound**	**Source^a^**	**Solvent**	**Purity (%)**
Acacetin	1	Ethanol	97
Allyl disulfide	1	Methanol	80
Apigenin	1	Ethanol	95
Apigenin 7-glucoside	1	Water	97
Baicalein	1	Ethanol	98
Baicalin	1	Acetone	95
Camphor	1	Methanol	98
Chelerythrine chloride	1	Water	95
Cichoriin	1	Methanol	98
*p*-Coumaric acid	1	Ethanol	100
Cyanidin chloride	2	Methanol	100
*p*-Cymene	1	Methanol	99
Daidzein	1	Methanol	98
Delphinidin chloride	1	Methanol	95
Embelin	1	Methanol	98
Emetine	1	Methanol	97
Eucalyptol	3	Methanol	99
Flavone	1	Acetone	100
(-)-Gallocatechin	1	Methanol	98
Genistein	2	Ethanol	99
α-Glycyrrhetinic acid	1	Methanol	98
β-Glycyrrhetinic acid	1	Methanol	97
Hyperforin	1	Methanol	85
Inulin	1	Water	100
Lavandulol	3	Methanol	90
(*S*)-(-)-Limonene	1	Methanol	96
Myrcene	3	Dichloromethane	90
Myricetin	2	Ethanol	100
Naringenin	1	Ethanol	100
Pelargonidin chloride	1	Ethanol	100
Piceatannol	4	Ethanol	98
ß-Sitosterol	1	Methanol	99
Sorgoleone	5	Ethanol	95
Stigmasterol	1	Methanol	99
Vitexin	1	Methanol	96
Wogonin	1	Ethanol	98
Zingiberone	1	Ethanol	96

^a^ Sources are as follows: (1) Sigma-Aldrich, St. Louis, Missouri, USA; (2) Indofine Chemical Company, Hillsborough, New Jersey; (3) Fluka Analytical, Buchs, Switzerland; (4) A.G. Scientific, San Diego, California; (5) Dr. Franck Dayan, USDA-ARS-NPURU, University, Mississippi.

For determination of 24-h 50% inhibition concentration (IC50) and minimum inhibition concentration (MIC), sterile 96-well polystyrene microplates (Corning Costar Corp., Acton, Massachusetts) with flat-bottom wells were used to conduct the bioassay for compounds that were dissolved in 100% ethanol, 100% methanol, or double-deionized water. In order to prevent solvent interaction with the polystyrene, sterile 96-well quartz microplates (Hellma Cells, Inc., Forest Hills, New York) were used for compounds dissolved in acetone or dichloromethane. Dissolved test compounds were added to microplate wells (10 µL/well). Solvents were allowed to completely evaporate before standardized bacterial culture (0.5 MacFarland) was added to the microplate wells (200 µL/well). For compounds dissolved in water, 10 µL/well of dissolved compound were added, and 10 µL/well of double-deionized water were added to respective control wells. Microplates were incubated at 29 ± 1 °C (VWR model 2005 incubator; Sheldon Manufacturing, Inc., Cornelius, Oregon). A Packard model SpectraCount microplate photometer (Packard Instrument Company, Meriden, Connecticut) was used to measure the absorbance (630 nm) of the microplate wells at time 0 and 24 h.

In order to determine the minimum bactericidal concentration (MBC), microplates were inspected visually after the 24-h incubation period for the growth assay, and 5 µL of culture material was aseptically transferred from microplate treatment wells with observed growth inhibition onto 3.8% MH agar plates for *E. ictaluri* and onto modified Shieh agar plates for both genomovars of *F. columnare*. All agar plates were incubated at 29 ± 1 °C for 3–5 days. Plates were then inspected for growth, and the MBC was determined to be the lowest concentration for which no bacterial growth was observed on the agar surface.

The means and standard deviations of absorbance measurements were calculated, graphed, and compared to controls to help determine the 24-h IC50 and MIC for each test compound [12]. The 24-h IC50 and MIC results for each compound tested were divided by the respective 24-h IC50 and MIC results obtained for the positive controls florfenicol and oxytetracycline to determine the relative-to-drug-control florfenicol (RDCF) and relative-to-drug-control oxytetracycline (RDCO) values.

## 4. Conclusions

Organisms in the kingdom Plantae provide a vast number of compounds that have so far been relatively unexplored for antibacterial activity, especially against *E. ictaluri* and *F. columnare*. In this study, a rapid bioassay was used as the first step to identify plant compounds with potential for use as therapeutants against the common disease-producing bacteria responsible for causing ESC and columnaris in pond-raised channel catfish. Among the compounds evaluated, chelerythrine chloride and ellagic acid were the most toxic toward *E. ictaluri*. Chelerythrine chloride, ellagic acid, β-glycyrrhetinic acid, sorgoleone, and wogonin were the most toxic compounds toward the isolates *F. columnare* ALM-00-173 and *F. columnare* BioMed. Prior to conducting any efficacy studies, the water-solubility characteristics of these active compounds and the potential toxicity towards non-target organisms will need to be considered.
